# To What Extent Should Porcelain Displace Gold in the Dental Art?

**Published:** 1904-05

**Authors:** J. Q. Byram

**Affiliations:** Indianapolis.


### TO WHAT EXTENT SHOULD PORCELAIN DISPLACE GOLD IN THE DENTAL ART?

By J. Q. Byram, Indianapolis.

Porcelain teeth were introduced in France about 1808. About 1825 M. Planton, a Frenchman, came to Philadelphia, bringing with him some porcelain teeth, such as were used in his native country. Samuel W. Stockton (an uncle of Samuel W. White), a jeweler by trade, who was very much interested in ceramics, became interested in the teeth and soon produced some that were far superior to the French product. It is said that Mr. Stockton carried his formulæ "in his head," and when he ceased to manufacture teeth, he refused to divulge his secret. So those who engaged in the manufacture of artificial teeth at this time were compelled to pass through a long experimental period. But after years of constant endeavor, there has been such an improvement in porcelain that today we are not only able to procure from the manufacturer teeth that are artistic and strong, but porcelain bodies, which enable the dentist to practice dental ceramics with a marked degree of success.

Dr. Charles H. Land says:* "Porcelain as applied to dentistry prior to the year 1885 was exclusively confined to artificial teeth, and a few cavity stoppers. The dawn of the new era which revealed a field of greater scope and greater value was first made known to the profession by the publication of an article in the Independent Practitioner of August, 1886, entitled 'A New System of Restoring Badly Decayed Teeth.'"

With the birth of the new century began the development of the dental ceramic art with such great enthusiasm and improvement that no up-to-date dentist can practice artistic dentistry without adding porcelain to the list of filling materials. If porcelain is to displace gold in the dental art, we must determine to what extent it may be used with safety. For an~ material tried so long as gold, cannot be cast aside for a comparatively new one unless it can be demonstrated beyond a doubt that the new material will do what is claimed for it.

Ceramics, as applied to the dental art, may be classified into: (1) The products of the manufacturer, and under this should be classed all forms of porcelain teeth (including crowns) and porcelain bodies. (2) The products of the dentist. This may be divided, first, into the replacement of lost teeth, as in dentures and bridges; second, the restoration of the entire crown of the tooth; third, the restoration of a portion of a crown, as in a porcelain tip or an inlay.

The difficulty of construction and the expense of a continuous gum denture cause it to be a denture for the few. It is to be regretted that more platino-porcelain bridges are not constructed. They are especially indicated in spaces between the cuspids. If the superior incisors have been extracted for a length of time and there is a sinking of the lip, it is quite impossible to restore this condition with a gold-porcelain bridge. A porcelan surface

*Dental Cosmos, June, 1903.

is far superior to gold for mastication, so in all cases where the length of the teeth will permit of a large enough mass of porcelain for strength, platino-porcelain bridges between cuspids and molars may be used. In spaces between cuspids and molars in the inferior arch, there are but few cases where the gold-porcelain bridge with the occlusal surface of gold, has advantages over the all-gold bridge, and unless a bridge with the occlusal surface of porcelain is constructed the all-gold bridge should be inserted.

If there is any branch of dentistry in which porcelain can almost displace gold, it is in crowns. To say that all teeth should be crowned with porcelain is just as absurd as to say all teeth should be filled with porcelain. There is a great deal said about educating the public to a higher appreciation of artistic dentistry, but until we educate the dentist in esthetics we cannot hope for rapid progress. At a recent dental meeting there were at least fifteen dentists persent, with gold crowns adjusted on incisors and cuspids. Imagine one of them teaching esthetic dentistry. There are so many forms of platino-porcelain and gold-porcelain crowns, that there is no excuse for adjusting gold crowns on incisors, cuspids and bicuspids, except in cases of excessive abrasion and where the teeth are quite short.

One criticism offered to the platino-porcelain crown is that the platinum shows at the cervical margin, or in course of time, it may cause the gum to have a bluish tinge. If caps are constructed by the following method, there will be no platinum exposed on the labial or buccal surfaces, and no opportunity for the gum to turn blue. Prepare the root so that opposite walls are parallel. Grind the face of the root flat, and rootward to the margin of the gum in the middle third. Take a wire measurement and fit a platinum band to the root. Grind the labial one-third of the root and band concave, mesio-distally, following the curvature of the gingival line. Remove the band and tack the lingual portion to the floor with the smallest amount of platinum solder. Adjust the band to the root and burnish the floor to it. This allows the labial one-third of the cap to follow the curvature of the gingival line, and if the labial portion of the face of the root has been ground just beneath the gum margin, the entire labial portion of the band will be covered by the gum. In case of recession of the gum, or where it is deemed advisable to expose the metal on the labial or buccal surface, the platinum may be covered with pure gold by placing small pieces on the surface to be covered, and fusing in the furnace.

As a filling material, porcelain is attracting the universal attention of the profession at the present time. And to what extent it will displace gold, depends upon the skill of the dentist and his clientele. There is a diversity of opinion of the practicability of porcelain as a general filling material, and may of the best operators seem skeptical about its advantages over gold. Some of the porcelain enthusiasts have done more to cause well founded skepticism than the careful, conservative advocates can remove in a decade.

Dr. W. T. Reeves goes so far as to say: *"There are scarcely any cavities where gold can be utilized that porcelain cannot be successfully used, and there are so many places and conditions under which gold cannot be used, and where porcelain will be a perfect filling material with which to restore the lost tooth structure, that I place it first in the point of applicability." In the same article he says: "After the cement is completely crystallized, a thin porcelain filling in the occlusal surface of a molar will have the full strength of the whole tooth to resist the masticating stress and, there is no danger of fracture."

Dr. Reeves, in speaking of the retention of porcelain inlays, says: **"I believe inlays depend for their strength of retention upon the close adaptation and crystallization of cement under pressure. If two pieces of glass are adapted with water between them to exclude the air, one cannot forcibly pull them apart. A joiner prepares the surfaces of two pieces of wood so that they are in close adaptation to each other, and, placing glue between them, clamps them in a vise and allows it to

---

*Dental Review, May, 1902.

**International Dental Journal, July, 1903.

harden. If there be any appreciable amount of glue, there wil be no strength of joints. There is no strength in the glue. The strength is due to close adaptation and crystallization under pressure. If this be true, there is no need of inflicting the additional pain upon the patient that the self-retentive style of cavity formation would entail."

Such statements from enthusiastic experts will tend to get porcelain in ill repute, for the inexoerienced will follow the teaching of the experts and his judgment wil be misguided by their teachings. Many operations fail, not because of lack of skill of the operator, but on account of poor judgment and misinformation. Only a few days ago, a dentist of reputation as an operator, brought a matrix for an inlay to be baked. The cavity was prepared in such a manner that all walls converged toward the pulpal wall and instead of the walls forming angles, they formed curves. When asked how he expected the inlay to be retained, his reply was, "By the cement." (A case of misguided judgment.) It is fallacy to say that inlays can be made to fit cavities so tight that their adaptation can be compared to two pieces of glass rubbed together or to two pieces of wood glued together.

Dr. C. N. Johnson, one of the safest teachers to follow, says of inlays: *"The demand for porcelain inlays sprang from the indiscriminate disolay of gold in the anterior part of the mouth, whereby the esthetic sense of the people was too frequently offended. If the profession had more carefully studied the possibilities of platinum and gold for harmonizing shades on exposed surfaces, there never would have developed the reflection upon the dental art that has been so justly urged against it, but it still remains true that there are certain cavities in which a well-made porcelain inlav is more artistic in appearance than it is possible to attain with any metal filling. It is also true that inlay work is less exhausting to the patient than extensive filling-building, and these two considerations should induce every operator to so perfect himself in inlay work that he is enabled to give his patients the benefit of the highest class of skill in those cases where inlays are indicated."

"The desirability of controlling caries in the anterior part of the mouth without the necessity for an objectionable display of gold, as also would the possibility of saving badly decayed teeth in any location where the insertion of gold foil is contra-indicated on account of too great tax on the patient or too much infliction on the impaired peridental membrane by the use of the mallet. Crown work as a result of the conditions just indicated, has too often been resorted to by operators in cases of extended decay at a period earlir than would make crowning more justifiable if some more feasible means could be employed to tide the tooth over a number of years. It is in cases of this kind that inlay work finds its most legitimate field."

It seems that the inexperienced would obtain better results in inlay work to follow the suggestions of the conservative teachers and not be carried away with the idea that they can use porcelain as a universal filling material because a few experts do. The indications for porcelain inlays have been discussed so much of late that every dentist who reads the journals has some idea of their application.

All labial and buccal cavities can be filled with porcelain to a better advantage than with gold. The patient is relieved of the unpleasantness of adjusting the rubber dam, the excruciating pain caused by the use of cervical clamps, and the sensitivity to thermal changes. Inlays can be made for such cavities that will harmonize with the natural teeth to such a degree that only the expert can detect them.

To what extent porcelain may be used in simple approximal, approximo-incisal and approximo-occlusal cavities, depends largely on stress. It is doubtful whether an approximal incisal or an approximo-occlusal cavity can be so prepared, and an inlay so constructed that it will resist stress to the same extent that a gold filling will. The friability of porcelain prevents the step portion of the filling from adding as much resistance as an equal mass of gold. Gold is a better filling material than porcelain unless a large portion of the occlusal surfaces of bicuspids and molars is in-

---

*Principles and Practice of Filling Teeth, 2d Edition.

volved. It is impossible to construct thin occlusal inlays without thin margins which are liable to fracture.

Those cases involving the incisal one-third or more of incisors and cuspids may be restored with porcelain by combining the prnciples of inlays and crowns. In case of a malformed incisor or cuspid, a porcelain tip can be adjusted to the tooth which will give as good service as a gold tip and better than a gold filling, and from the esthetic point of view it has every advantage over gold. The occlusal portion of bicuspids and molars may be restored with porcelain, but unless enough of the tooth is involved to make a large mass, there will be insufficient strength in the porcelain to withstand the force of mastication.

The fact that inlays are held in position with cement has prevented many operators from placing confidence in them. The permanency of inlays depends on their close adaptation to the cavity walls, for it has been found that the solution of the thin film of cement around the inlay is only for a slight depth, leaving the cavity sealed so that there seems to be no recurrence of decay.—*American Dental Journal.*

